# Nursing during World War II: Finnmark County, Northern Norway

**DOI:** 10.3402/ijch.v72i0.20278

**Published:** 2013-04-25

**Authors:** Ingrid Immonen

**Affiliations:** Department of Health Science, Finnmark University College, Hammerfest, Norway

**Keywords:** Nursing, history, World War II, Finnmark County

## Abstract

**Introduction:**

This study is part the project “Nursing in Borderland – Finnmark 1939–1950” within nursing history that sheds light on nursing and health care during World War II in Finnmark County, Northern Norway. The study focuses on challenges in nursing care that arose during the war because of war activities in the Barents area. This article focuses on challenges caused by shortage of supplies. The aim of the project is to widen the understanding of development within health care and living conditions in the area.

**Study design:**

This is a historical study using narratives, government documents and literature.

**Methods:**

Interviews with nurses and persons active in health care during World War II constitute the main data of the research. Thematic issues that arise from interviews are analysed.

Primary and secondary written sources are used in analysing the topics.

Because of war activities, deportation and burning of the county, archives were partly destroyed. Central archives can contribute with annual reports, whereas local archives are fragmentary. There are a number of reports written soon after the War, as well as a number of biographical books of newer date.

**Results:**

Challenges caused by war, which appear in the interviews, are: 1) shortage of supplies, 2) increased workload, 3) multicultural society, 4) ethical dilemmas, 5) deportation of the population. In this paper, focus is on challenges caused by shortage of supplies.

**Conclusions:**

Both institutions, personnel and patients were marked by the war. This has to be taken in consideration in health care today.

World War II had a big impact on all of Europe. There were a number of frontiers, communities were ruined by war activities, and foreign forces occupied several countries. Norway was one of the countries occupied by Germany. Although there were only minor battles within Norway, the sea passages of Northern Norway, past Troms and Finnmark Counties and through the Barents Sea to the Soviet Union, were important for transport of supplies to Allied forces.

Documentation on health services in Finnmark County during the war is scarce. This is partly due to destruction from shelling and the “scorched earth” tactics towards the end of the War. The archives that remain are fragmentary, and there is little work done on health of the civilian population.

During the post-war reconstruction period, health care in Finnmark County was rebuilt. It was modelled largely on the pre-war system, though with improvements due to advances in the health sciences. Research on how the civilian population coped with war challenges is limited. There are Ph.D. and Master's theses on nurses in war (1,2), but these focus on nurses working as volunteers in conflict areas or as drafted nurses. Ruud (3), and more recently Kinnunen and Jokisipilä (4) have studied how memories of wartime develop and change with time in the national context.

The project “Living the War” is a cooperative project involving nurses, historians and anthropologists. The aim of the project is to throw light on health care and living conditions for the civilian population during the War in the Barents region. This article, based on data from one of the subprojects, “Nursing in Borderland – Finnmark 1939–1950”, aims to analyse how health care personnel, especially nurses, coped with shortages of supplies during the period of occupation and war activities in Finnmark County.

## Finnmark County

Finnmark is the northernmost county in Norway. It has an area of 48,637 km^2^ and a population of approximately 73,000 (5). The county covers 15% of the Norwegian mainland but only 1.5% of the country's total population (6).

In Finnmark County, there is a long tradition of many cultures living together: Sami, Russian, Kven, Karelian, Finnish and Norwegian. Trade routes between Northern Norway and other parts of Scandinavia and Russia have been well established for centuries.

The borders in the northern part of Norway have been adjusted several times during the past centuries. From 1920, the eastern border was with the Petsamo region of Finland. The “Continuation War” was fought between Finland and the Soviet Union from June 1941 to September 1944. It ended with the Moscow Armistice in 1944 when Finland ceded Petsamo to the Soviet Union. Norway thus acquired its northern border with the Soviet Union at the end of World War II (7).

### Health care in Finnmark

Because of long distances and the sparse population, people in Finnmark have largely depended on their own resources for daily life and have developed the ability to find practical solutions. This also applies to health care.

At the outbreak of the War, there were 4 hospitals in the county – 3 situated in the eastern part (Vadsø, Vardø and Kirkenes) and one in the west (Hammerfest). Kirkenes hospital was a private hospital run by the mining company for its employees and families, but it also treated local residents. In addition to municipalities, several non-governmental organisations were involved in institutions for the elderly, orphans and tubercular patients, as well as in home-based nursing and small local hospitals (8). For a long period, “the Asylum” in Hammerfest served as a psychiatric nursing home for the entire county (9).

During the War, there was no national framework in Norway for nursing education. The Norwegian Nurses Association (NSF) campaigned for a law requiring 3 years of education for all nurses. This law was not passed until 1948 (10). Nurses were recruited to hospitals and district nursing through nursing organisations such as the Red Cross, Deaconesses and the Norwegian Women's Public Health Association. In addition, Hammerfest Hospital trained 6 nurses in a 3-year programme. The title “nurse” was not only used by professional nurses with a 3-year education – assistant nurses and sanitary personnel with a shorter duration of training who worked in health care were also addressed as “sister” or “nurse” (10).

### Finnmark and World War II

Finnmark County held a strategic position during the War, as Allied supplies were transported through the Barents Sea to northern parts of the Soviet Union, a lifeline that the German army was keen to interrupt. There were battles across the German–Soviet Litsa frontier on the Kola Peninsula for a long period before the Soviet army finally moved into the eastern parts of Finnmark in October 1944. During the War, a large number of German soldiers were stationed in Finnmark. In some municipalities, there were 10 times as many German soldiers as local inhabitants. The German army requisitioned private homes, schools and other institutions as offices, housing, hospitals, and so on (11). Much action occurred at sea, in the air and on the ground. Kirkenes in the east was one of Europe's most heavily bombed cities during the War, and in many municipalities the damage caused by shelling was considerable (12).

At the beginning of the War there was a small army sanitary unit in Finnmark. This was transferred to Nordland County during hostilities at Narvik in April 1940 (13). For the remainder of the war, the civil authorities, NGOs and the civilian population provided health care. In addition, the German army had sanitary stations in the county, and they also commandeered hospital wards and other facilities for use by the German army.

## Methods

### Interviews

Interviews were made during 1998–2011 with nurses, nuns and nurse-helpers who served in Finnmark during the War and the post-War reconstruction period, and represent both hospital and district nursing in the county.

Most nurses who were asked to take part in interviews were positive and found it important to tell their stories. A small number did not want to talk about the war period. It is difficult to know in what way analyses of the material would differ if these persons had participated (14).

Interviews were made as open interviews with a focus on the everyday work during the War. There was a guide for questions on certain topics such as shortage of food and medical supplies and equipment, and communications, but mainly it was my role to elicit nurses’ stories on topics that they thought important. Interviews were transcribed and analysed focusing on significant/meaningful issues. In analysing interviews, one should bear in mind that narratives are already analysed by the narrator in a cultural context and later experiences to confirm personality and social standing (15).

Ruud has, in his Master thesis “Memories of World War II in Finnmark and Northern Troms” (3), shown how memories tend to shift with lapse of time after the war. The first period after a war tends to be patriotic, exemplified by the heroic stories of those who did withstand the orders from central war authorities to evacuate the county, and who stayed behind during the scorch earth tactics from the withdrawing German forces. When peace had stabilised the country again, memories become more nuanced, but they were also influenced by the political situation in the country. He also shows how some trends are common for the country as a whole, and how memories and memorials differ in northern part of Norway from southern parts, due to differences in war experiences. Kinnunen and Jokisipilä (4) find a similar pattern in Finnish war memories.

### Written materials

Written sources and archives were used as complementary materials to provide a deeper understanding and to validate data from interviews.

There is little contemporary written material concerning civilian health care in Finnmark during the War. What is left in archives, both national and local, is fragmentary. This is mainly due to war damages in the eastern parts of the county, and to restrictions in the amount of personal effects that refugees could carry with them when they were deported under the “scorched earth” policy in the western parts of the county.

Central archives contain annual medical reports from all districts, summing up the staffing situation, changes in bed capacity, and the main health issues during the year – births, deaths, epidemics and vaccinations, together with general reflections on the population's health condition.

Other important sources include *Sykepleien*, the periodical of the Norwegian Nurses Association (NSF), during the period 1935–1950. Archives from the Norwegian News Agency (NTB) during the war period offer an insight into media restrictions. This is valuable knowledge when reading newspapers from the period and assessing their contents.

Immediately after the War, there were a number of articles, pamphlets and books written on experiences during the War, notably Axel Strøm's study of the nutrition situation (16), and the Kirkenes fire brigades’ recollections of their war experiences (12).

During the past 10–20 years, there has been a renewed interest in war history and a number of articles have been written in local historical books, novels for children and adults as well as historical books and text books (11,17–19). There are also a number of Master's and doctoral theses (1,3,20).

## Results

All municipalities in Norway had their own offices for stock supplies and rationing that were coordinated by county offices. The municipal offices were in charge of ordering stock, obtaining transport and maintaining a ration system. Rations were equal in all parts of Norway during the War, but the ability to buy rationed food and other stock varied in different parts of the country, and also with the seasons (21).

### Transportation

A large amount of supplies were transported to Finnmark by boat, as there was no railroad in the north, and roads were of poor quality. As the sea-lanes were heavily mined and attacked from the air and at sea, delivery of medicines and food supplies, as well as building materials, clothing and shoes, was unreliable, delayed and often lost altogether (11).

Unreliable transportation also affected recruiting new staff and getting relief for staff. As many nurses had their families in the southern part of Norway, leaving Finnmark for family visits became difficult. During peace time, travel routes northwards were by train, bus and boat through Norway, a trip that could be done in about a week. During the War, one had to take the route through Sweden to Rovaniemi in Finland, and onwards by bus or car to Finnmark, with unreliable schedules and improvised accommodation. As some informants recalled:

There was no room in the hotel, so I had to sleep in the bath tub; The driver went boozing, so we could not go the next day; There was this lorry going north. One of the men knew the driver, so we could catch a ride on the back of the lorry. (Interview A)

### Food supplies

Most nurses in Finnmark, both hospital and district nurses, had free board and lodging as part of their remuneration. Hospitals also experienced shortages in food supplies. Nurses describe the situation at Hammerfest hospital as:

Food was ordinary. Of course there was little of anything and the bread could smell of cat's piss. (Interview B)

Daily butter rations for the staff were the size of a thumb fingernail. Flour was often of poor quality and difficult to make proper bread with. Sometimes patients could get extra rations in addition to hospital diet from their families. One year there was a cook at the hospital who could get hold of a large portion of stockfish through private connections. This influenced the menu all through winter.

On weekends, the German wards at Hammerfest hospital received special food from their own army kitchen in town. The craving for food was so strong that hospital staff sometimes sneaked some food from these extra supplies.

This was of course risky and one had to be quite sure not to get caught. But the extra treat was worth the risk. (Interview B)

Those who had cattle were best off, as they provided both milk and meat. It was common to share what scarce supplies one had. This family of 9 persons had a cow and a calf:

One day we discovered that someone was stealing our calf. We ran after the thieves, they let go of the calf and we caught it. We saw three men in poor clothes vanishing in the woods on the other side of the lake. When we went home Mummy said: we should have let them have the calf, they needed it more than us. But then we could not reach them anymore. They were escaped Russian prisoners. (Interview C)

In rural areas, the water sources were wells and sometimes small rivers and lakes.

Our teeth went bad. It was the water. We took water from the little lake in the marsh. So did the German soldiers who lived in the cottage in the neighbourhood. They also got bad teeth. Then their officer took water samples from the lake and there was a lot of iron in it, and they stopped using it. They got their water from a brook. We were a big family, and it was too far to bring the amount of water we needed from there, so we still took our water from the lake. And all the children got bad teeth. (Interview C)

There was also a shortage of vitamins. Potatoes were an important source of vitamin C and had, for the most part, to be imported from other parts of the country. Potatoes transported to the county by boat could be drenched with salty seawater or even frozen on deck before they reached their destination (Annual medical reports 1940, 1941).

District doctors recorded in their annual reports the lack of potatoes and milk as important causes of low immunity in the population and, as such, one of the reasons for the population generally being in poor health (Annual medical reports 1940, 1941). Pregnant women especially were marked by a lower intake of calcium, iron and vitamin C. “One child, one tooth” was a common saying in Finnmark well into the 1970s.

In mid-January 1943, one of the nurses in Porsanger reported:

Børselv ran out of flour a couple of weeks ago, but on the west-side of the fjord it is much worse, as they have been out of flour much longer. Around Christmas there was a ship with potatoes, the westside and Børselv got potatoes, but when the ship reached Hamnbukt, frost destroyed the whole load, and so Lakselv, Karasjok, Kjæs and Brenna didn't get any potatoes. (National Archives Tromsø 1941)

Fish, as an important part of the local diet, was difficult to get in most parts of the county as waters were mined, and it was risky to go fishing. Fresh water fish was available though in many parts of the county.

### Shortage in medicines and medical equipment

Shortages of medical supplies meant that the nurses had to improvise. Bandages had to be washed and reused. After accidents with many casualties, such as wrecking of fishing boats, there was a great need for bandages within a short time. Nurses sometimes asked youths from town to help with tearing up old and worn linen for bandages.

Antibiotics were not yet in common use. Sulphanilamide and sulphapyridin were available. The latter was also called “M&B 693” as it was produced in the laboratories of May and Bakers (22).

Due to the scarcity of supplies, difficult decisions often had to be made as to who should get the needed medicine and who should not. One nurse recalled a conversation with a doctor:

He (the doctor) put a syringe and bottle in my hand and said “The Red Cross-guys are right there with the car (the ambulance and crew). You'll go with them”, he said, “there is a man with diphtheria. Give him as much as he needs of what you have in the bottle, if he's not too poorly. Then you don't give him anything, because we don't have much medicine, and we need to be careful with what we have”. “But how do I know how much to give him, then?” I asked, “I haven't worked at the epidemics ward”. “Ooh” The doctor pulled his hair, he was upset and said: “Are you a nurse or are you not?” (Interview D)

## Discussion

Even though the context differs between my research and that of Virtanen (1) and Tjoflåt (2), it is relevant to compare my findings with theirs. Both Virtanen and Tjoflåt analyse nurses’ experiences from the perspective of preparedness for work in conflict areas. In Finnmark nurses’ work was within the frames of the civilian population, without any special preparedness for work in conflict.

We tend to look at phenomena in terms of dichotomies: war–peace, sick–healthy, enemy–friend. My study shows that everyday life comprises a variety of nuances. In war time, this becomes especially clear and shows the challenges that nurses had to cope with regarding patriotism, nursing ethics, theoretical and experience-based knowledge in a war context.

Virtanen (1) points at some unifying values that nurses experience during war: professionalism, love for one's neighbour, religiosity and patriotism. My informants show these values in their stories, as they transform them into practical experiences in daily life.

While different parts of Finnmark County had different war experiences, there were common challenges in coping with shortage of supplies and poor transportation. All nurses who worked in Finnmark during the War were part of the civilian society and thus influenced by local conflicts and how these were met in everyday life in a community with a majority of residents from the occupying forces.

After the war nurses report long-term health consequences in the population, as increased alcoholism and “nerves”. Finne (20) shows in his theses how these long-term consequences also affect the life of later generations.

Tjoflåt's informants, who served for defined periods in conflict areas, report little or no psychological stress after their term in conflict areas. Preparing for the duty through teaching programs, however short, seems to be important, also debriefing after homecoming. During the War, nurses in Finnmark had no special preparation for the challenges they faced but were required to continue their work as nurses.

As Ruud (3) points out, there is not always congruence between the official memory about the War and personal recollections. Kinnunen and Jokisipilä (4) highlight the changes that occur in official memory of war events with lapse of time and also with changes in the political situation. This is also put into words by my informants who were relieved that one could tell the story as it was:

It has only lately become proper to say openly that it was the Russians who freed Finnmark. (Interview E)

Nurses had to use their imagination, act independently and use all their physical and mental strength during this period. Not only were working hours long in an ordinary work situation, they also had to stay on duty for extra time, as with casualties from shipwrecks and bombing raids. They had to use what supplies were available, sometimes old linen or even toilet paper for bandages.

Just like the rest of the civilian population, nurses faced food rations and difficult lodging situation, and were thus exposed to unhealthy conditions that often led to illness. Many nurses were exhausted and happy to step back when they could: “You are young, it is your turn now”.

Nurses who worked in Finnmark and Northern Norway during the war were often misunderstood both during and after the war. Already during the war, there was a view in the southern parts of the country that northerners were too friendly with the enemy. Nurses had to put their personal views aside when nursing German patients. It seems that nursing ethics was considered in everyday work, placing the patient in focus. Regarding injured German soldiers, a nurse said, “They were just young boys”.

## Concluding remarks

Why should knowledge of these historical experiences of nurses in war times be of interest to health care professionals in the 21st century? One reason is that how patients understand illness and health care is shaped by the past experiences, whether personal or passed on to them by earlier generations. The oldest generation in Finnmark is still marked by their war experiences. To know about these experiences is important to understand them better and meet their needs.

In the reconstruction period (until the 1960s), health care in the county was rebuilt largely on basis of its earlier experiences with decentralised health care. Nurses and other health personnel shaped by such experiences helped influence the directions of health care. Norway has recently implemented a new Norwegian Public Health Act (23,24). In planning these changes, the Ministry of Health and Care Services did look to the north for a coordination model to develop nationwide. As a region with a sparsely distributed population, Finnmark County has practised interagency and multi-sectoral cooperation among governmental, municipal and private health and care institutions for a long time.

Many nurses were exhausted after the war and found it difficult to resume their work, resulting in a nursing shortage during the reconstruction period. Married nurses began entering the workforce in this period. For my informants, it was important to tell their story, as they say there are only losers in a war. Even the soldiers are “just ordinary boys”, and the civilian population also paid a high price in terms of their physical and mental health.
